# Regional Gene Expression of LOX-1, VCAM-1, and ICAM-1 in Aorta of HIV-1 Transgenic Rats

**DOI:** 10.1371/journal.pone.0008170

**Published:** 2009-12-04

**Authors:** Anne Mette Fisker Hag, Ulrik Sloth Kristoffersen, Sune Folke Pedersen, Henrik Gutte, Anne-Mette Lebech, Andreas Kjaer

**Affiliations:** 1 Cluster for Molecular Imaging, Faculty of Health Sciences, University of Copenhagen, Copenhagen, Denmark; 2 Department of Clinical Physiology, Nuclear Medicine and PET, Rigshospitalet, Copenhagen, Denmark; 3 Department of Infectious Diseases, Hvidovre Hospital, Hvidovre, Denmark; BMSI-A*STAR, Singapore

## Abstract

**Background:**

Increased prevalence of atherosclerotic cardiovascular disease in HIV-infected patients has been observed. The cause of this accelerated atherosclerosis is a matter of controversy. As clinical studies are complicated by a multiplicity of risk-factors and a low incidence of hard endpoints, studies in animal models could be attractive alternatives.

**Methodology/Principal Findings:**

We evaluated gene expression of lectin-like oxidized-low-density-lipoprotein receptor-1 (LOX-1), vascular cell adhesion molecule-1 (VCAM-1), and intercellular adhesion molecule-1 (ICAM-1) in HIV-1 transgenic (HIV-1Tg) rats; these genes are all thought to play important roles in early atherogenesis. Furthermore, the plasma level of sICAM-1 was measured. We found that gene expressions of LOX-1 and VCAM-1 were higher in the aortic arch of HIV-1Tg rats compared to controls. Also, the level of sICAM-1 was elevated in the HIV-1Tg rats compared to controls, but the ICAM-1 gene expression profile did not show any differences between the groups.

**Conclusions/Significance:**

HIV-1Tg rats have gene expression patterns indicating endothelial dysfunction and accelerated atherosclerosis in aorta, suggesting that HIV-infection *per se* may cause atherosclerosis. This transgenic rat model may be a very promising model for further studies of the pathophysiology behind HIV-associated cardiovascular disease.

## Introduction

The morbidity and mortality among patients with human immunodeficiency virus (HIV) infection has declined dramatically since the introduction of antiretroviral therapy (ART). Concomitantly, increased rates of atherosclerotic cardiovascular disease in HIV-infected patients have been observed. However, the cause of accelerated atherosclerosis behind most cardiovascular diseases, developed by HIV-patients, is still a matter of controversy. Three possibilities have been suggested: the infection itself, the treatment used or the dyslipidemia often seen in HIV-patients [1], [2]. The clinical studies are limited by the fact that the patients are influenced not by one but many factors, including different cardiovascular risk-factors, *e.g.* smoking. As it is not possible to totally isolate the different causes in clinical experiments, an animal model represents an attractive alternative to investigate in detail the origin of accelerated atherosclerosis associated with HIV-infection *per se*.

A transgenic rat model of HIV was constructed in 2001 (the HIV-1Tg rat) based on a Sprague-Dawley x Fisher 344/NHsd F1 background [3]. The HIV-1Tg rat contains a *gag*-*pol*-deleted HIV-1 provirus regulated by a viral promoter. Several experiments have shown this rat model to display clinical manifestations resembling the manifestations seen in HIV-patients. These manifestations include wasting, skin lesions, cataract, neurological signs, and respiratory difficulty. Furthermore, the cardiac pathology seen in the HIV-1Tg rat resembles the pathology seen in HIV-patients [3]–[6]. To our knowledge, development of atherosclerosis has not previously been investigated in the HIV-1Tg rat or any other HIV animal model.

The first stage of atherosclerosis is considered to be endothelial dysfunction [7], [8]. As we want to investigate atherosclerosis in HIV-1Tg rats, although rats do not normally have this disease [9], we have chosen to focus on targets that are characteristic of the earliest stage of atherosclerosis. The three targets used in this study are: lectin-like oxidized-low-density-lipoprotein receptor-1 (LOX-1), vascular cell adhesion molecule-1 (VCAM-1), and intercellular adhesion molecule-1 (ICAM-1).

Oxidized low-density lipoprotein (oxLDL) is important in the initiation and development of atherosclerosis. The primary endothelial receptor of oxLDL, LOX-1, is responsible for binding, internalization, and degradation of oxLDL in endothelial cells. Both *in vitro* and *in vivo* experiments have shown the receptor to be important in the initiation of atherosclerosis and to be up-regulated by pro-atherogenic factors, *e.g.* shear stress, tumor necrosis factor (TNF)-α, and oxLDL itself [10], [11].

VCAM-1 and ICAM-1 also play important roles in the process of atherosclerosis as they are vital to leukocyte attachment, rolling, and trans-endothelial migration. VCAM-1 is primarily an inducible molecule, whereas ICAM-1 is also constitutively expressed on resting endothelial cells. They are both up-regulated by pro-atherogenic factors. VCAM-1 has, based on experiments with knockout mice, been recognized as the crucial adhesion molecule in the initiation of atherosclerosis [12], [13]. A high level of soluble ICAM-1 (sICAM-1) in plasma has been linked to increased risk of cardiovascular events, as well as to cancer and autoimmune diseases [14].

The aim of the present study was to look at regional gene expression of LOX-1, VCAM-1, and ICAM-1 in the aorta of HIV-1Tg rats to identify possible signs of accelerated atherogenesis caused by HIV. Furthermore, the plasma level of sICAM-1 was measured.

## Results

### Gene Expression

**Figure 1:**
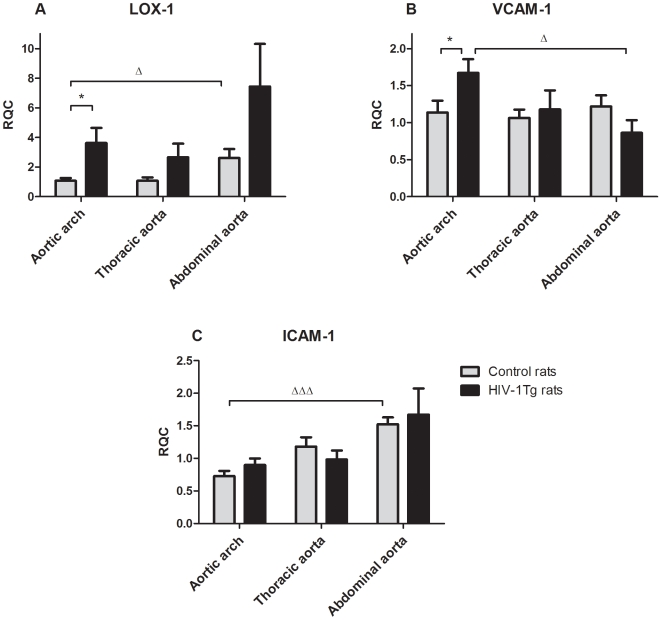
Gene expression relative to the calibrator (RQC) expressed as mean ±SEM of *n* = 8–12. (A) LOX-1: *p<0.05 aortic arch, HIV-1Tg vs. control rats; ^Δ^ *p*<0.05 control rats, aortic arch vs. abdominal aorta. (B) VCAM-1: *p<0.05 aortic arch, HIV-1Tg vs. control rats; ^Δ^ *p*<0.05 HIV-1Tg rats, aortic arch vs. abdominal aorta. (C) ICAM-1: ^ΔΔΔ^ *p*<0.001 control rats, aortic arch vs. abdominal aorta.

Gene expression of LOX-1 (Fig. 1A) was significantly up-regulated in the aortic arch of HIV-1Tg rats compared to control rats (3.4-fold, *p* = 0.041). A tendency to LOX-1 up-regulation in the thoracic (*p* = 0.13) and abdominal aorta (*p* = 0.14) compared to the control rats was also present, although non-significant.

Gene expression of VCAM-1 (Fig. 1B) was significantly up-regulated in the aortic arch of HIV-1Tg rats compared to the control rats (1.5-fold, *p* = 0.044). No differences were found in the thoracic or abdominal aorta between HIV-1Tg and control rats. In HIV-1Tg rats the gene expression level for VCAM-1 was higher in the aortic arch compared to the abdominal aorta (1.9-fold, *p* = 0.011).

There were no significant differences in ICAM-1 gene expression between HIV-1Tg and control rats (Fig. 1C).

### Plasma Level of sICAM-1

**Figure 2:**
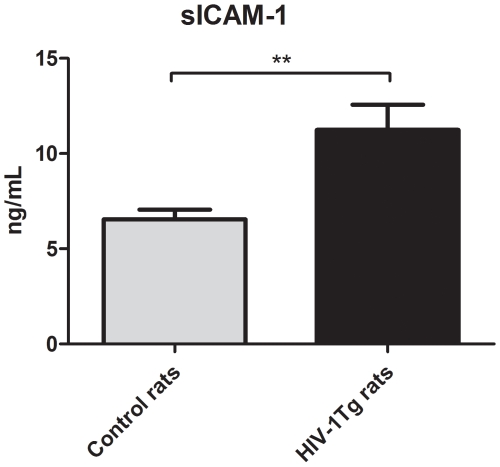
The plasma level of sICAM-1 expressed as mean ±SEM of *n* = 8–12. **p<0.01 HIV-1Tg vs. control rats.

The plasma level of sICAM-1 is shown in Fig. 2.

sICAM-1 was significantly increased in the HIV-1Tg rats compared to the control rats (1.7-fold, *p = *0.009). The range of sICAM-1 was 4.3–17.2 ng/mL.

## Discussion

Here we show for the first time that gene expression of LOX-1 and VCAM-1 are up-regulated in the aortic arch of HIV-1Tg rats compared to control rats. In contrast, the gene expression of ICAM-1 was not signficantly up-regulated. Although the HIV-infected rats did not display overt atherosclerosis, the up-regulation of LOX-1 and VCAM-1 indicates early atherogenesis in these rats. Therefore, even though overt atherosclerosis is normally not seen in rats [9], our study indicates that early, pre-atherosclerosis can still be studied in HIV-1Tg rats. Accordingly, the HIV-1Tg rat-model seems suitable for further studies of development of accelerated atherosclerosis in HIV.

The up-regulation of LOX-1 and VCAM-1, but not ICAM-1 is in line with our previous study of pre-atherosclerotic apoE^−/−^ mice [15]. According to that study, ICAM-1 did not seem to play a role in atherogenesis as early as LOX-1 and VCAM-1. Another study using immunohistochemistry found VCAM-1 to be up-regulated in 20 weeks old apoE^−/−^ mice compared to control mice. The up-regulation of VCAM-1 was further increased by high-fat diet, whereas ICAM-1 was unaffected by both apoE-deficiency and high-fat diet [16]. These studies support that our findings in the present study are signs of early atherosclerosis.

The significant up-regulation of LOX-1 and VCAM-1 in the aortic arch but not in the other areas of the aorta is not unexpected. It has long been known that specific patterns of shear stress influence gene expression in the vessel wall. The initial observation was that atherosclerosis develops in a non-random pattern [17] . A study of aortas from normal adult pigs showed higher expression of pro-inflammation associated genes in the aortic arch than in the thoracic aorta. They concluded that regions with low shear stress (aortic arch) were more atherosclerosis-susceptible than regions with laminar flow and high shear stress (thoracic aorta) [18]. Numerous studies of cell cultures using different flow apparatuses have demonstrated the association between the hemodynamics of vessels and the expression of pro-atherosclerotic genes. A study from 2002 using human aortic endothelial cells showed VCAM-1 to be up-regulated by disturbed flow which is characteristic of the aortic arch. The expression of ICAM-1 was only transiently upregulated [19]. Based on both *in vitro* and *in vivo* experiments, the overall conclusion is that low, disturbed, or oscillating flow patterns promote a pro-inflammatory phenotype that paves the way for atherosclerosis [20], [21]. In accordance with this, we found indications of HIV-induced accelerated atherosclerosis in the aortic arch, where low and oscillating flow is present.

We found that sICAM-1 was significantly higher in HIV-1Tg rats compared to control rats. In accordance with our results, clinical studies have shown that sICAM-1 is up-regulated in HIV-patients compared to non-infected individuals [22], [23]. The origin of sICAM-1 is so far unknown [14]. The higher plasma level of sICAM-1 without upregulation of the gene expression of ICAM-1 in the aorta indicates that vessels may not be the major source of sICAM-1.

The relevance of specific HIV-proteins to the development of atherosclerosis has so far not been fully revealed. Until now the research calls attention to the HIV proteins Tat, gp120 and Nef as possible mediators of endothelial dysfunction [24]. As these proteins are expressed in all cells of the HIV-1Tg rat, this study cannot disclose their relevance for the disease. However, it could be an interesting future area of investigation to study the impact of these proteins on atherogenesis in an animal model.

In conclusion, HIV-1Tg rats have gene expression patterns indicating accelerated atherosclerosis in the aortic arch. This indicates that HIV-infection *per se* seems to cause atherosclerosis. The HIV-1Tg rat may be a promising model for further studies of the pathophysiology behind HIV-related cardiovascular disease, *e.g.* time-course of the atherogenesis and relative importance of HIV-infection and anti-retroviral treatment. Furthermore, it will be interesting to study how different risk-factors, *e.g.* high-fat diet, influence the HIV-related atherogenesis.

## Materials and Methods

### Experimental Model

Animal care and all experimental procedures were performed under the approval of the Danish Animal Welfare Council.

Eight male HIV-1 transgenic rats and 12 parental wild-type inbred F344/NHsd control background strain rats were purchased from Harlan (IN, USA).

The rats were housed under humidity-, temperature-, and light cycle-controlled conditions, and had free access to standard rat diet and water throughout the course of experiments.

### Sample Handling

At the age of 10–12 months, the animals were anesthetized with 3% sevoflurane (Abbott Scandinavia AB, Sweden) mixed with 35% O_2_ in N_2_ by breathing and subsequently decapitated. Blood was collected, centrifuged, and plasma transferred to a fresh tube and stored at −20°C.

The aorta was removed and divided into the aortic arch, the thoracic aorta, and the abdominal aorta. The samples were placed in RNAlater® (Ambion (Europe) Limited, UK) and stored at 4°C. The following day RNAlater® was removed and the samples were stored at −80°C.

### RNA Extraction and Reverse Transcription

Total RNA was isolated with TRI Reagent® (Molecular Research Center Inc., OH, USA), and 0.3 µg total RNA was reversed transcribed using the AffinityScript™ QPCR cDNA Synthesis Kit from (Stratagene, CA, USA, cat.# 600559). Samples were cooled down and stored at −20°C.

### Real-Time qPCR

Gene expression was quantified on the Mx3000P® and Mx3005P™ real-time PCR systems from Stratagene. LOX-1 was quantified in a duplex with glyceraldehydes-3-phosphate dehydrogenase (GAPDH), while VCAM-1, ICAM-1, and GAPDH were quantified in a triplex. The Brilliant® QPCR Core Reagent Kit (Stratagene, cat.# 600530) was used. Optimization of assays resulted in a 50% and 100% increase of dNTP and Taq polymerase for the duplex and triplex, respectively. An MgCl_2_ concentration of 5.5 mM was found to be optimal for all experiments. The thermal profile used was: 10 min of denaturation at 95°C followed by 45 cycles with denaturation for 30 s at 95°C and annealing/elongation at 60°C for 1 min.

Relative quantification by the comparative method (2^−ΔΔCt^) [25] was used. A calibrator consisting of a pool of cDNA from all control rats was used. Furthermore, the measurements were corrected for the efficiency of the PCR reaction, calculated from 5-fold dilution curves, thereby replacing 2 in the formula with (1+E).

The optimal housekeeping gene for the study was selected from a panel of twelve common endogenous control genes (pre-fabricated panel from PrimerDesign, UK). All 12 genes were tested by qPCR in both HIVTg and control rats. NormFinder [26] was used to find the optimal housekeeping gene. GAPDH was found to be the most representative housekeeping gene.

Primers and TaqMan dual-labeled probes for the three genes of interest LOX-1 (NM_133306), VCAM-1 (NM_012889), and ICAM-1 (NM_012967) along with the housekeeping gene GAPDH (NM_017008) were designed using Beacon Designer (PREMIER Biosoft, CA, USA). Primers and probes are shown Table 1. All primers and probes were purchased from Eurofins MWG (Ebergsberg, Germany).

**Table 1:** Primers and probes.

Name	Forward primer (5′–3′)	Reverse primer (5′–3′)	5′ fluoro-phore	Probe (5′–3′)	3′ quen-cher	Amplicon length (bp)
LOX-1	ttgcatactttgtagacagtctctc	ctgagtttcatccattttggtcatg	FAM	ttgaccctgccatgccatgctatga	BHQ-1	88
VCAM-1	tttgcaagaaaagccaacatgaaag	tctccaacagttcagacgttagc	CY5	ctgtgcctccaccagactgtacgat	BHQ-2	88
ICAM-1	agatcatacgggtttgggcttc	tatgactcgtgaaagaaatcagctc	FAM	ccacaggtcagggtgctttcctcaa	BHQ-1	75
GAPDH	ccgtgttcctacccccaatg	cttcaccaccttcttgatgtcatc	HEX	ctccaggcggcatgtcagatccac	BHQ-1	91

For each gene, the optimal primer and probe concentration was established (Table 2). All samples were run as triplicates using 1 µL of cDNA.

**Table 2:** Optimal filter setting, primer- and probe-concentration for the genes investigated.

Name	Forward primer-concentration, nM	Reverse primer-concentration, nM	Probe-concentration, nM	Filter settings
LOX-1	300	300	400	FAM ×4
VCAM-1	600	300	250	CY5 ×2
ICAM-1	300	300	300	FAM ×4
GAPDH	300	300	250	HEX ×2

### Plasma Measurements

All plasma samples were measured using a bead-based sandwich immunoassay technique [27], [28]. A commercially available kit (cat.# RCVD2-89K-01, LINCO Research, MO, USA) was used. The samples were analyzed on the Luminex™ LX100 with STarStation 2.0 software (Millipore Corporation, MA, USA).

### Statistical Analysis

All data were tested by means of Kolmogorov-Smirnov and found to be normal distributed. Comparisons between the different rat groups were performed by Student's *t*-test for independent samples and between regions of aorta with a paired *t*-test. Bonferroni corrections for planned comparisons were performed when more than two groups were compared. *P*<0.05 was considered significant.
